# A Useful Blood Flow Restriction Training Risk Stratification for Exercise and Rehabilitation

**DOI:** 10.3389/fphys.2022.808622

**Published:** 2022-03-11

**Authors:** Dahan da Cunha Nascimento, Nicholas Rolnick, Ivo Vieira de Sousa Neto, Richard Severin, Fabiani Lage Rodrigues Beal

**Affiliations:** ^1^Department of Physical Education, Catholic University of Brasília (UCB), Brasília, Brazil; ^2^Department of Gerontology, Catholic University of Brasília (UCB), Brasília, Brazil; ^3^The Human Performance Mechanic, Lehman College, New York, NY, United States; ^4^Laboratory of Molecular Analysis, Graduate Program of Sciences and Technology of Health, University of Brasília, Brasília, Brazil; ^5^Department of Physical Therapy, College of Applied Health Sciences, The University of Illinois at Chicago, Chicago, IL, United States; ^6^Department of Physical Therapy, Robbins College of Health and Human Sciences, Baylor University, Waco, TX, United States; ^7^Department of Nutrition, Health and Medicine School, Catholic University of Brasília (UCB), Brasília, Brazil

**Keywords:** blood flow restriction, risk assessment, risk factors, kaatsu, assessment

## Abstract

Blood flow restriction training (BFRT) is a modality with growing interest in the last decade and has been recognized as a critical tool in rehabilitation medicine, athletic and clinical populations. Besides its potential for positive benefits, BFRT has the capability to induce adverse responses. BFRT may evoke increased blood pressure, abnormal cardiovascular responses and impact vascular health. Furthermore, some important concerns with the use of BFRT exists for individuals with established cardiovascular disease (e.g., hypertension, diabetes mellitus, and chronic kidney disease patients). In addition, considering the potential risks of thrombosis promoted by BFRT in medically compromised populations, BFRT use warrants caution for patients that already display impaired blood coagulability, loss of antithrombotic mechanisms in the vessel wall, and stasis caused by immobility (e.g., COVID-19 patients, diabetes mellitus, hypertension, chronic kidney disease, cardiovascular disease, orthopedic post-surgery, anabolic steroid and ergogenic substance users, rheumatoid arthritis, and pregnant/postpartum women). To avoid untoward outcomes and ensure that BFRT is properly used, efficacy endpoints such as a questionnaire for risk stratification involving a review of the patient’s medical history, signs, and symptoms indicative of underlying pathology is strongly advised. Here we present a model for BFRT pre-participation screening to theoretically reduce risk by excluding people with comorbidities or medically complex histories that could unnecessarily heighten intra- and/or post-exercise occurrence of adverse events. We propose this risk stratification tool as a framework to allow clinicians to use their knowledge, skills and expertise to assess and manage any risks related to the delivery of an appropriate BFRT exercise program. The questionnaires for risk stratification are adapted to guide clinicians for the referral, assessment, and suggestion of other modalities/approaches if/when necessary. Finally, the risk stratification might serve as a guideline for clinical protocols and future randomized controlled trial studies.

## Introduction

Blood flow restriction training (BFRT) has been recognized as a critical tool in rehabilitation medicine, athletic and clinical populations. Although increases in muscle strength following high load resistance training (RT) appear significantly greater than low load RT with BFR, BFR induces similar hypertrophy and lower joint forces/stress with low load RT compared to high load traditional RT without BFR (Bagley et al., 2015; Scott et al., 2016; Hughes et al., 2017; Centner et al., 2019; Rolnick and Schoenfeld, 2020a). Besides the potential implementation of BFRT in clinical musculoskeletal rehabilitation (e.g., knee osteoarthritis, and anterior cruciate ligament reconstruction) (Hughes et al., 2017), clinicians prescribing BFRT are often faced with the BFRT paradox: while participation in regular BFRT (e.g., aerobic training, resistance training, and passively without exercise) is acknowledged to offer significant benefits in muscle mass and strength, it can possibly result in adverse events (e.g., numbness, nausea, hypertension, headache, venous thrombus, deterioration of ischemic heart disease, fainting, tingling, excessive pain, central retinal vein occlusion, and rhabdomyolysis) if applied inappropriately (Nakajima et al., 2006; Ozawa et al., 2015; Noto et al., 2017; Yasuda et al., 2017; Patterson and Brandner, 2018; de Queiros et al., 2021). Such occurrences are very infrequent but have been previously documented.

As the use of BFRT continues to expand in clinical practice, the available literature does not definitively answer if the positive health outcomes outweigh the risks of adverse signs, symptoms, or events during or after exercise with BFR. Nonetheless, despite the beneficial adaptations to skeletal muscle, little is known about long-term changes to vascular health and hemodynamics (Wong et al., 2021). This is important as most studies have stringent inclusion/exclusion criteria, leaving limited data on individuals with comorbidities frequently seen in rehabilitation clinics (Severin et al., 2020).

Despite the desirable effects on skeletal muscle function, BFRT may evoke increased blood pressure and abnormal cardiovascular responses secondary to the augmented and sustained activation of the muscle metaboreflex (Spranger et al., 2015; Cristina-Oliveira et al., 2020). Furthermore, some important concerns with the use of BFRT exists for individuals with established cardiovascular disease (e.g., hypertension, diabetes mellitus, and chronic kidney disease patients), as even appropriate use of BFRT could lead to clinical deterioration of vascular health caused by increased retrograde shear stress, intermittent sympathetic overactivity, and blood pressure elevation (Domingos and Polito, 2018; Wong et al., 2018; da Cunha Nascimento et al., 2020a,b). Thus, these potential adverse outcomes do not support the general claims about safety of BFRT for medically compromised populations (e.g., chronic disease and under cardiac rehabilitation) (Spranger et al., 2015; Cristina-Oliveira et al., 2020).

Conversely, considering the risks of thrombosis promoted by BFRT, a recent systematic review demonstrated that BFR exercise does not exacerbate the activation of coagulation nor enhance fibrinolytic activity (Nascimento et al., 2019). However, it isn’t easy to advocate that BFRT is innocuous due to the state of the current literature from the heterogeneity of applied BFRT protocols. BFRT should be prescribed with caution, especially for medically compromised populations or those with increased risk of clotting (e.g., anabolic agents).

To avoid untoward outcomes and ensure that BFR exercise is properly used according to current best practice guidelines, efficacy endpoints such as a questionnaire for risk stratification involving a review of the patient’s medical history, signs, and symptoms indicative of underlying pathology is strongly advised. A recent review paper reported that a significant barrier to successful BFRT implementation includes difficulty with integrating a comprehensive and systematic medical screening process, and determining when it is best to include BFRT into a plan of care while considering relevant participant characteristics like pain, loading intolerances, clotting issues, hemodynamics, and recent physical activity history (Rolnick et al., 2021). The proposed model aimed to improve the provision of best practice BFRT prescription for people across the health spectrum by utilizing a thorough screening process. However, the screening approach did not mention specific medical diagnoses frequently encountered in clinical practice and instead focused on encouraging pertinent thought processes likely to minimize risk when applying BFRT in medically compromised populations. Therefore, the proposed approach lacks specificity in assigning relative safety risk profiles to medically compromised populations commonly seen in outpatient rehabilitation and thus the assessment of risk is still primarily left to clinician opinion.

Evaluation of the individual patient for BFRT represents a potentially complex medical screening problem. A list of individual risk factors that are associated with adverse responses to BFRT has already been discussed and proposed elsewhere to aid in the screening process (Nakajima et al., 2011; Kacin et al., 2015; Brandner et al., 2018; Bond et al., 2019; Rolnick et al., 2021). However, a considerable amount of information regarding their impact on health is scattered throughout the literature and not compiled in a BFR-specific resource. A screening tool that considers the available evidence on BFRT that relies on a comprehensive personal, medical, and family history will assist clinicians in proposing the best management strategy for an individual patient with a given condition. We believe this approach will minimize the risk of adverse events while maximizing health benefits during BFRT.

Therefore, the overall aim of this manuscript is to provide a potentially useful BFRT questionnaire for risk stratification for exercise and rehabilitation. We review the primary adverse and beneficial effects of BFRT for healthy individuals and populations with chronic disease including hypertension, cardiovascular disease, rheumatoid arthritis, diabetes mellitus, and chronic kidney disease as well as assessing potential risks in patients following COVID-19 infection, post-surgery, those who are pregnant or postpartum and individuals with or without use of anabolic steroids, and ergogenic substances. Furthermore, we provide additional insights into the application, effectiveness, and utilization of BFRT for different populations while discussing future directions that warrant consideration in basic and clinical studies. These reports have high relevance in the field of exercise physiology and sports medicine as BFRT is a rapidly growing modality in fitness and rehabilitation settings.

## Developing a Risk Assessment Tool in Chronic Disease

Clinicians are encouraged to consider this risk stratification when exercising their judgment in determining and implementing BFRT with their patients. This pre-screening tool does not supersede the responsibility to make appropriate and accurate decisions in consideration of each patient’s health condition and in consultation with the patient and their referring physician and/or other members of their healthcare team.

A questionnaire for risk stratification to BFRT can theoretically optimize safety and mitigate risk. We recommend considering the patient’s baseline health status and tailoring guidance accordingly. Some may not exercise with BFR without risk. Interventions with lower risk must be encouraged when BFRT is deemed unsafe, or the risk outweighs potential benefits.

These recommendations herein aim to minimize the risk of adverse events in high-risk patients with comorbidities or other conditions that may decrease the safety profile of BFRT. However, it is essential to recognize that most exercising populations engage in leisure time physical activity with minimal negative acute or long-term outcomes. Unlike leisure time physical activity, BFRT likely needs some degree of supervision to minimize risk. Supervision by knowledgeable clinicians should theoretically reduce the occurrence of adverse events (e.g., numbness, nausea, hypertension, headache, venous thrombus, deterioration of ischemic heart disease, fainting, tingling, excessive pain, central retinal vein occlusion, and rhabdomyolysis) (Nakajima et al., 2006; Ozawa et al., 2015; Yasuda et al., 2017; Patterson and Brandner, 2018; de Queiros et al., 2021) especially when performed under those who adequately screen out high risk patients and use recommended guidelines (Patterson et al., 2019; Rolnick and Schoenfeld, 2020a,b; Rolnick et al., 2021) to structure exercise programming.

The clinician should contemplate some specific questions before the application of BFRT:

• Is my patient like the participants in the studies with BFRT?
• Does BFRT have a clinically relevant benefit (e.g., improved function or hypertrophy) that outweighs the potential risks of application?
• Is another treatment or method available that could provide similar results with less risk than BFRT?

For the risk assessment tool, previous recommendations and guidelines in chronic disease were adapted (Fletcher et al., 2001; Milech et al., 2016; Diabetes, 2019; Mach et al., 2020; Pelliccia et al., 2021). Also, exclusion criteria from small clinical studies with BFRT were integrated. Something important to address is that BFRT practitioners (e.g., physiotherapy, physical education teacher, or strength and conditioning coaches) are required to have a necessary physiological and pathoanatomical background knowledge on these conditions to apply the proposed risk stratification. Due to the judgment required and lack of any formal credentialing processes for those engaging in disseminating BFRT knowledge to clinicians and other providers, physician consultation may be necessary to clarify complex medical problems or their severity (Rolnick et al., 2021).

## Thrombosis Risk and Blood Flow Restriction Training

A recent systematic review concluded that exercise with BFR does not exacerbate the activation of coagulation or enhance fibrinolytic activity (Nascimento et al., 2019). However, the current body of literature with respect to risk of thrombosis resulting from BFRT does not completely exclude the potential for deep vein thrombosis (DVT) formation. A recent study evaluated the feasibility of BFRT (>125% of arterial occlusion pressure, AOP) in patients with incomplete spinal cord injury and increased risk for DVT (Stavres et al., 2018). After 4 days of the experimental protocol, participants had blood drawn for D-dimer analysis. The D-dimer cut off values are normally < 500 ng/mL and medically ill subjects whose D-dimer is elevated beyond this value constitute a subgroup with high risk of first DVT occurrence, DVT recurrence, and mortality in which prospective evaluation is necessary (Halaby et al., 2015). Participants who displayed an abnormally high quantitative D-dimer (>500 ng/mL) underwent a second bilateral leg ultrasound. Two showed an elevated D-dimer (>500 ng/mL) after 4 days of the experimental session, but no DVT was observed (Stavres et al., 2018). Of note, this study utilized pressures not recommended in clinical practice (125% AOP) (Patterson et al., 2019).

It is important to mention a case report of Paget-Schroetter Syndrome (PSS) after an acute BFRT session (Noto et al., 2017). PSS is an idiopathic subclavian vein thrombosis due to compression at the thoracic outlet. Prior to DVT formation, the individual reported suffering from localized edema on the left collarbone with mild tenderness for 6 years. When performing BFRT (30 min to 1 h, three times a week) she became aware of additional swelling, pain and discoloration of her left upper limb. Blood tests revealed a slight increase in her D-dimer (1.7 μg/mL) level and subsequently was diagnosed with PSS derived from thoracic outlet syndrome. Physicians suspect that stagnation of the blood flow due to pressure applied during BFRT, venous retraction and endothelial dysfunction of the left subclavian vein had likely caused the PSS. Also, another case study reported an adverse effect of BFRT on a patient with diabetic retinopathy and a central retinal vein occlusion that was preceded by a session of BFRT (Ozawa et al., 2015).

Nonetheless, there appears to be the potential for DVT formation in those that may have medically complex histories. Thus, concerns with the use of BFRT in medically compromised individuals is relevant (Table 1). In addition, a limited number of studies have measured other hemostatic markers to determine safety issues for DVT necessitating additional pre-screening to theoretically enhance safety (Nascimento et al., 2019). Markers such as antithrombin deficiencies, protein C, cofactor protein S, and homocysteine may help further stratify potential risk for BFRT in those with comorbidities (Motykie et al., 2000; Caprini et al., 2004).

**TABLE 1:** Summarizes concerns about the use of BFRT associated with DVT development in medically compromised populations.

Medical condition	Concerns about use of BFRT and DVT
Hypertension	• Patients with hypertension are in a hypercoagulative and potentially prothrombotic state. This increased thrombotic risk has primarily attributed to the endothelial dysfunction associated with hypertension. • Additionally, hypertension frequently presents with elevations in hemostatic factors such as P-selectin (platelet aggregator), fibrinogen, and PAI-1 (Yang et al., 2010).
Post-COVID-19 infection	• Patients with COVID-19 may develop both venous and arterial system coagulopathy caused by endotheliitis, hypercoagulopathy, and stasis (Sarkar et al., 2021).
Pregnancy/Postpartum women	• Pregnancy has been shown to result in elevations in fibrinogen, factor VII, factor VIII, von Willebrand factor, factor IX, factor X, factor XII, and PAI-1 which increases the risk of DVT formation (Prisco et al., 2005). • Other factors such as delivery method (cesarean section) and obesity, multiparity, and medical comorbidities increases the risk for DVT as well (Alsheef et al., 2020). • Following pregnancy, DVT risk is 5 times higher, and acquisition of a pulmonary embolism is 15 times more likely than during pregnancy (Heit et al., 2005). • Increases in pro-coagulant and decreases in anti-coagulants are observed in OCP users compared to non-users (Gunaratne et al., 2021).
Diabetes mellitus	• Patients with DM type 1 or type 2 are at increased risk of DVT due to systemic changes and endothelial dysfunction (Diabetes, 2019). • Hyperglycemia triggers vascular damage by an imbalance between nitric oxide (NO) and reactive oxidative species (ROS), platelet aggregation, inflammation, and increased expression of coagulant tissue factors like PAI-1 (Paneni et al., 2013; Kaur et al., 2018).
Rheumatoid arthritis and Chronic kidney disease	• Rheumatoid arthritis patients are at an elevated risk of VTEs, pulmonary embolisms and DVT formation compared to the general population (Li et al., 2021). • Elevated thrombogenic factors can help explain the excessive risk for CVD, and all appear in a greater proportion of CKD patients than the general populace (Levey et al., 1998).
Post-surgery	• The risk of DVT is increased 100-fold in the first 6 weeks following surgery (Bond et al., 2019) and pulmonary embolism risk is more significant in the 12 weeks following surgery in middle-aged women, which of course, will depend on the type of surgery (Sweetland et al., 2009). • The relative risk for thrombosis after hip and knee arthroplasty is 220 times higher in the first 6 weeks after surgery, 91.6 times higher after cancer surgery, and 87 times higher after vascular surgery highlighting that surgery of any kind increases risk of DVT formation (Sweetland et al., 2009).
Anabolic steroid users and certain ergogenic aids*	• Users have a high risk of suffering from thrombotic complications, cardiomyopathy, stroke, pulmonary embolism, fatal and non-fatal arrythmias, and myocardial infarction (Sculthorpe et al., 2012). • Anabolic steroid users have side effects such as dyslipidemia, polycythemia, hyperhomocystemia, hypercoagulability state, cardiac and vascular hypertrophy, impaired angiogenesis, redox imbalance, and cardiomyocyte apoptosis (Seara et al., 2020).

BFRT, blood flow restriction training; DVT, deep vein thrombosis; VTE, venous thromboembolism; CVD, cardiovascular disease; OCP, oral contraception; PAI-1, plasminogen activator inhibitor-1; CKD, chronic kidney disease. *Anabolic/ergogenic agents are not considered a medically compromised population but exhibit heightened risk for negative vascular sequalae that predispose to DVTs.

The following sections will introduce relevant background information and pre-screening processes regarding patients with diabetes mellitus, cardiovascular disease and hypertension, rheumatoid arthritis, chronic kidney disease, COVID-19 infection and those post-surgery as well as those patients who engage in the use of anabolic steroids, ergogenic substances, are pregnant/postpartum and those who are apparently otherwise healthy.

## Thrombosis Risk Assessment Before Blood Flow Restriction Training

Considering the above-mentioned presentations that likely may be at an elevated risk of DVT formation secondary to a prothrombotic state, risk stratification for DVT is strongly encouraged to be included during the initial screening process prior to BFRT (Prisco et al., 2005; Yang et al., 2010; Paneni et al., 2013; Kaur et al., 2018; Gupta et al., 2020; Seara et al., 2020). The adapted thrombosis risk factor assessment from Caprini (Motykie et al., 2000; Caprini, 2005; Golemi et al., 2019) represents a useful tool and was previously used in BFRT studies as exclusion criteria (Loenneke et al., 2013, 2015; Jessee et al., 2018; Table 2). It may be possible that the Caprini risk assessment model is too stringent, and for a patient with a risk factor score of five (e.g., hip fracture), BFRT might still be a preferrable method for rehabilitation given medical clearance and a reasonable amount of recovery time has passed (Motykie et al., 2000; Caprini, 2005; Golemi et al., 2019). Nonetheless, the use of the International Medical Prevention Registry on Venous Thromboembolism (IMPROVE) (Spyropoulos et al., 2011; Mahan et al., 2014; Rosenberg et al., 2014; Raskob et al., 2016) that incorporates seven well established and easy-to-implement clinical risk factors for DVT could be used when clinicians consider the Caprini risk assessment model (Motykie et al., 2000; Caprini, 2005; Golemi et al., 2019) not applicable for anamnesis.

**TABLE 2:** Thrombosis risk factor assessment.

Patient’s Name:________________________________________________________________________ Age:_________________________________________________________________________________ Sex:_________________________________________________________________________________			

Each risk factor represents 1 point	Each risk factor represents 2 points		
□ Abnormal pulmonary function (COPD); □ Acute myocardial infarction; □ Age between 41 and 59 years; □ Blood transfusions; □ Chemotherapy; □ Congestive heart failure (<1 month); □ Diabetes requiring insulin; □ History of inflammatory bowel disease; □ History of prior major surgery (<1 month); □ Length of a surgery > 2 h; □ Medical patient currently on bed rest; □ Minor surgery planned; □ BMI > 25–39; □ Obstructive pulmonary disease; □ Sepsis (<1 month); □ Serious lung disease including pneumonia (<1 month); □ Smoking; □ Swollen legs (current); □ Varicose veins; □ Other risk factors: easy bruising, for example, must be included as may represent a platelet disorder (Ballas and Kraut, 2008);	□ Age 60–74 years; □ Arthroscopic surgery; □ BMI > 40; □ Central venous access; □ Immobilized plaster cast (<1 month); □ Laparoscopy surgery (>45 min); □ Major surgery (>45 min); □ Malignancy (present or previous); □ Patient confined to bed (>72 h);		

**Each risk factor represents 3 points**	**Each risk factor represents 5 points**		

□ Age over 75 years; □ Any acquired congenital thrombophilia; □ Elevated anticardiolipin antibodies; □ Elevated serum homocysteine; □ Family history of thrombosis; □ Heparin-induced thrombocytopenia; □ History of DVT/PE; □ Positive Factor V Leiden; □ Positive lupus anticoagulant; □ Positive Prothrombin 20210A; □ If the answer is yes: □ Type:___________________________	□ Acute spinal cord injury (paralysis) (<1 month); □ Elective major lower extremity arthroplasty; □ Hip, pelvis or leg fracture (<1 month); □ Multiple trauma (<1 month); □ Stroke (<1 month);		

**For women only (each represents 1 point)**	**Total risk factor score**		
□ History of unexplained stillborn infant, recurrent spontaneous abortion (≥3), premature birth with toxemia, or growth-restricted infant; □ Oral contraceptives or hormone replacement therapy; □ Pregnancy or postpartum (<1 month);	**Score**	**Incidence of DVT**	**Risk level**

	0–1	<10%	Low
	2	10–20%	Moderate
	3–4	20–40%	High
	5 or more	40–80% and risk of mortality of 1–5%	Highest

*Adapted from Caprini (2005). DVT, deep vein thrombosis; PE, pulmonary embolism; BMI, body mass index; COPD, chronic obstructive pulmonary disease.*

Using the IMPROVE risk assessment model, patients are classified into low-risk tier (0–1 points), moderate-risk tier (2–3 points), and high-risk tier (≥4 points) (Table 3; Spyropoulos et al., 2011; Mahan et al., 2014; Rosenberg et al., 2014; Raskob et al., 2016).

**TABLE 3:** Modified IMPROVE risk score.

Patient’s Name:______________________________________________________________________________ Age:________________________________________________________________________________________ Sex:________________________________________________________________________________________	

DVT risk assessment	DVT risk score
Previous VTE	3
Known thrombophilia^a^	2
Current lower leg paralysis or paresis^b^	2
Prior cancer^c^	2
ICU/CCU stay	1
Complete immobilization ≥ 1 day^d^	1
Age ≥ 60 years	1

*ICU, intensive care unit; CCU, cardiac care unit; VTE, venous thromboembolism. ^a^A congenital or acquired condition leading to excess risk of thrombosis (e.g., factor V Leiden, lupus anticoagulant, factor C or factor S deficiency). ^b^Leg falls to bed by 5 s, but has some effort against gravity or presence hemiparesis, hemiplegia, paraplegia, and quadriplegia. ^c^Cancer (excluding non-melanoma skin cancer) present at any time in the last 5 years (cancer must be in remission to meet eligibility criteria). ^d^Confined to bed or chair with or without bathroom privileges. Adapted from previous studies (Spyropoulos et al., 2011; Mahan et al., 2014; Rosenberg et al., 2014; Raskob et al., 2016).*

For individuals who are hypertensive, post-COVID-19 infection, post-surgery, have rheumatoid arthritis, chronic kidney disease, cardiovascular disease, diabetes mellitus or engage in anabolic agents/steroids and/or ergogenic substances, we recommend the use of Caprini or IMPROVE scales (Caprini, 2005; Rosenberg et al., 2014) for thrombosis risk assessment in addition to normal pre-screening processes.

Of note, use of BFRT during or closely following pregnancy/post-partum period is a contentious topic with regards to safety and potential DVT risk. Only one case study has been reported during pregnancy. The case report applied BFRT in the third trimester (1 set of 30 repetitions followed by 20 repetitions and then 15 repetitions, a rest interval of 20 s between sets, and with a setting pressure between 40 and 200 mmHg) using biceps curl with a 1 kg load with no negative influence on the fetal status and uteral-placental circulation (Takano et al., 2013). Despite the observations of no effect of BFRT on the female and the fetus, health professionals should exhibit caution when screening a female pregnant/postpartum prior to BFRT for thrombosis risk (Motykie et al., 2000; Caprini et al., 2004; Caprini, 2005). Considering that BFRT diminishes venous return, it is vital to understand the pathogenesis of DVT in pregnancy. Pregnant women have a 50% reduction in venous leg flow that begins to normalize by 6 weeks post-partum (Macklon and Greer, 1997; Brown and Hiett, 2010). Uteral growth impedes inferior vena cava and iliac vein flow, producing obstruction, increases in venous capacitance and blood stasis; all of which contribute to an elevated DVT risk (Brown and Hiett, 2010). As women usually become prothrombotic in pregnancy and the risk of DVT is higher, Caprini or IMPROVE scales (Caprini, 2005; Rosenberg et al., 2014) should not be used for this population.

Clinicians should consistently evaluate the pregnant patient for signs of venous thromboembolism (VTE) such as swelling and shortness of breath, changes in skin temperature, presence of tachycardia, pain or discoloration, and swollen or distended varicose veins in the affected limb (O’Brien et al., 2018). A simple clinical tool that has been used practically by the authors in other at-risk populations is taking periodic photographs of the affected limb every ∼4 weeks. If varicosities are increasing, it is strongly advised to discontinue BFRT.

Last, clinicians should be aware of May-Thurner syndrome (estimated prevalence of at least 20% in the general populace) during screening (Peters et al., 2012). May-Thurner syndrome occurs when the right iliac artery compresses the left iliac vein, predisposing the patient to iliac vein thrombosis and painless unilateral leg swelling (Golemi et al., 2019).

## Diabetes Mellitus and Blood Flow Restriction Training

A recent review study cited possible theoretical positive benefits of BFRT in patients in both type 1 (Jones et al., 2021) and type 2 diabetes mellitus (DM) (Saatmann et al., 2021). Satoh (2011) showed beneficial effects of BFRT on 51 cases with metabolic syndrome, as evidenced in a 10% drop in systolic blood pressure (SBP) and diastolic blood pressure (DBP), a 10% drop in HbA1c levels, an 8% decrease in LDL cholesterol and a 10% weight loss with no adverse events reported. Unfortunately, the study did not include a control group, limiting the ability to extrapolate whether the observed effects were from the exercise, the BFRT, or both. Nonetheless, the results support the use of BFRT in this population with clinically relevant improvements in hemodynamics and relevant metabolic syndrome markers. In addition, a recent study on rats with DM displayed that BFRT plus electrical stimulation prevented diabetes-associated muscle atrophy, highlighting that muscular responses to BFR exercise can be elicited despite the impaired systemic changes (albeit with evoked electrical stimulation) (Tanaka et al., 2019).

Recently, only one experimental study displayed that low-intensity BFRT resistance exercise compared to high-intensity resistance exercise in females with DM type 2 induces thrombocytosis (excessive number of platelet count and plateletcrit) but with similar platelet activation markers to high-intensity resistance training (Fini et al., 2021). This acute response might demonstrate the safety and potential useful of BFRT in this population compared to traditionally recommended approaches to strength training.

However, considering the low level of evidence in this particular population, we have only one study with DM patients (Fini et al., 2021). In the presence of any of the following risk factors cited below (Table 4), the patient with DM is classified as high risk (Fletcher et al., 2001; Milech et al., 2016; Diabetes, 2019; Mach et al., 2020), precluding the use of BFRT without physician clearance.

**TABLE 4:** Relevant risk factors concerning DM patients before beginning BFRT.

Patient’s Name:_____________________________________________________________________ Age:______________________________________________________________________________ Sex:_______________________________________________________________________________
□ *Patients with diabetes mellitus without organ damage with DM duration ≥ 10 years or another additional risk factor^a^; □ *Type 1 diabetes mellitus of long duration (>20 years); □ *Premature family history of cardiovascular disease^b^; □ *Presence of metabolic syndrome (Alberti et al., 2009)^c^; □ *Untreated systemic arterial hypertension; □ *Current smoker^d^; □ *Diabetes mellitus with target organ damage*^e^*; □ *Cardiovascular autonomic neuropathy; □ *Diabetic retinopathy; □ *Glycemia > 250 mg/dL with or without ketosis prior exercise; □ *Coronary calcium score > 10 Agatstone; □ *Carotid plaque (intima-media thickness > 1.5 mm); □ *Angiotomography of the coronary arteries with the presence of plaque; □ *Ankle-brachial index < 0.9; □ *Presence of abdominal aortic aneurysm; □ *Acute coronary syndrome; □ *Ischemic stroke or transient ischemic attack; □ *Peripheral vascular insufficiency (ischemic ulcer); □ *Revascularization of any artery for atherosclerosis: carotid, coronary, renal, and lower limbs; □ *Non-traumatic amputation of lower limbs; □ *Severe atherosclerotic disease with obstruction > 50% in any artery; □ Acute systemic illness; □ Angina or ischemic ST depression at a workload < 6 METs; □ Cardiomyopathy with ejection fraction < 30%; □ Complex ventricular arrhythmias not well controlled; □ Congenital heart disease; □ Coronary revascularization (percutaneous coronary interventions, coronary by-pass graft surgery, and other arterial revascularization procedure); □ Exercise capacity < 6 METs; □ Fall in systolic blood pressure below resting levels during exercise; □ Familial hypercholesterolemia with atherosclerotic cardiovascular disease or with another major risk factor; □ Marked elevated single risk factors, in particular total cholesterol (>310 mg/dL), LDL-C (>190 mg/dL), or blood pressure ≥ 180/110 mmHg; □ Multivessel coronary disease with two major epicardial arteries having 50% of stenosis; □ Myocardial infarction and unstable angina; □ Non-sustained ventricular tachycardia with exercise; □ Peripheral artery disease; □ Previous episode of primary cardiac arrest (e.g., cardiac arrest that did not occur in the presence of an acute myocardial infarction or during a cardiac procedure); □ Self-reported easy bruising (Ballas and Kraut, 2008); □ Stable angina; □ Stroke; □ Transient ischemic attack; □ Valvular heart disease with severe and asymptomatic valvular stenosis or regurgitation; □ SBP ≥ 160 mmHg and/or DBP ≥ 100 mmHg prior to exercise; □ Other medical condition that could be aggravated by exercise; □ A medical problem that the physician and BFRT-user believe may be life-threatening;

^a^Valid for individuals with ≥ 18 years. ^b^Presence of cardiovascular disease in a first-degree relative (only father, mother, or siblings) before aged 55 (men) and under 65 (women). ^c^Waist circumference ≥ 94 cm for men and ≥ 102 cm for women; elevated triglycerides (≥150 mg/dL) or drug treatment for elevated triglycerides; HDL-C < 40 mg/dL in males and < 50 in females or drug treatment for reduced HDL-C; SBP ≥ 130 mmHg and/or DBP ≥ 85 mmHg or drug treatment for elevated blood pressure; fasting glucose ≥ 100 mg/dL or drug treatment for elevated glucose. ^d^At least 1 year without smoking or similar. HDL, high density lipoprotein. ^e^Retinopathy, neuropathy, left ventricular hypertrophy; Carotid artery intima-media thickness > 0.9 mm or carotid plaque; Carotid-femoral pulse wave velocity > 10 m/s; Ankle-brachial index < 0.9; Stage 3 chronic kidney disease; Albuminuria between 30 and 300 mg/24 h or albumin-creatinine ratio urinary 30–300 mg/g. SBP, systolic blood pressure; DBP, diastolic blood pressure. *, risks are specific for diabetic patients. Adapted from previous studies (Fletcher et al., 2001; Milech et al., 2016; Diabetes, 2019; Mach et al., 2020; Pelliccia et al., 2021).

Diabetes mellitus patients display elevated levels of plasma homocysteine, soluble endothelial protein receptor (sEPCR) and high sensitivity C reactive proteins (hsCRP) signaling a pro-inflammatory state (van Guldener and Stehouwer, 2002; Zaghloul et al., 2014). Hyperglycemia on endothelial cells closely mimics that of inflammatory initiators (Funk et al., 2012). Furthermore, in DM type 1, sEPCR is associated with duration of the disease and hsCRP is associated with duration of disease and hypertension (Zaghloul et al., 2014). These changes have marked effects on fibrin structure-function, generating a denser clot with greater resistance to fibrinolysis (Grant, 2007). Factors such as decreased NO availability, oxidative stress (ROS imbalances), smooth muscle cell proliferation, increased leukocyte adhesion, enhanced platelet aggregation, and impaired fibrinolysis also increase DVT risk (van Guldener and Stehouwer, 2002; Zaghloul et al., 2014). Furthermore, considering that DM may remain undetected for many years and its diagnosis is often made incidentally, clinicians may encounter this disease at an advanced stage when vascular complications have already occurred (Beckman et al., 2013).

As DM is associated with a prothrombotic state (Paneni et al., 2013; Kaur et al., 2018), risk stratification for DVT (Tables 2, 3) in conjunction with the previous risk factors must be included (Table 4). However, clinicians should use clinical judgment, knowledge of risk factors, and experience to provide protection to the patient along with consulting other clinician experts/physicians when unsure of risk (Rolnick et al., 2021).

If the clinician decides to proceed with BFRT, frequent blood pressure monitoring during exercise sessions (e.g., during the exercise bout or during the rest periods between sets) is strongly recommended until safety is established (2–4 weeks) for those at moderate-to-high risk of cardiac complications during exercise (Fletcher et al., 2001). Further, when exercise positions are changed (e.g., seated leg extensions to standing squats), monitoring of blood pressure responses should be performed as the hemodynamic responses to exercise will likely differ (Hughes et al., 2018). Similarly, clinicians working with a patient with DM may also apply routine diabetic screening precautions. These include insulin checks, possibly evaluating ketone levels, verifying recent episodes of hypoglycemia, carbohydrate intake before and after exercise and monitoring for post-exercise hypoglycemia depending on professional scope of practice (Milech et al., 2016; Adolfsson et al., 2018; Diabetes, 2019).

## Hypertension, Blood Pressure, Heart Rate Variability, Angiogenesis and Blood Flow Restriction Training

Concerns about BFRT were raised previously on the effect of exercise with BFR when compared to exercise without BFR on hemodynamic and endothelial function. Shear stress is an important factor for inducing endothelial adaptation during exercise and is a major stimulus for NO release from the endothelium (Phillips et al., 2015). However, acute application of BFRT has exhibited lower shear stress and higher retrograde shear stress post-BFRT, suggesting a blunted reactive hyperemic response (da Cunha Nascimento et al., 2020b). Another study reported no changes in endothelial function or worsening changes when compared to the non-cuffed arm, highlighting the potential for attenuated local vascular adaptations with chronic BFRT exercise protocols (Tinken et al., 2010).

Some studies report acute reductions in flow-mediated dilation (FMD) (an essential index of endothelial function) for upper limbs (radial artery) and lower limbs (popliteal artery) with long-term reduction of FMD for upper limbs when compared to exercise without BFR (Credeur et al., 2010; Renzi et al., 2010; Tinken et al., 2010; Paiva et al., 2016). Thus, low shear stress and increased retrograde shear stress promoted by cuff use may contribute to endothelial dysfunction (Thijssen et al., 2009). Further support for potential endothelial dysregulation came from a previous study who reported increased DBP and mean arterial pressure response after a chronic low load BFRT protocol (Kacin and Strazar, 2011). These results may be of particular concern to individuals with hypertension as they show significantly greater SBP and DBP response compared to normotensive subjects during BFRT (Domingos and Polito, 2018).

A recent meta-analysis demonstrated that regular BFRT exercise elicits a significant 4.2 mmHg SBP increase over time (Wong et al., 2021). While small, those results may evoke safety concerns in those populations whose exercise pressor reflex may be altered such as in those with hypertension, heart failure, and peripheral arterial disease (Spranger et al., 2015; Cristina-Oliveira et al., 2020). However the findings from that meta-analysis were limited to a low number of included studies (4 studies) and a lack of a sub-group analysis taking into consideration absolute occlusion pressure, cuff width and occlusion pressure prescription (e.g., personalized vs. arbitrary values) on hemodynamic outcomes. Furthermore, a previous systematic review and meta-analysis demonstrated that values of SBP and DBP were significantly higher for hypertensive individuals compared to normotensive individuals during BFRT (Domingos and Polito, 2018). Nonetheless, as BFRT generates exaggerated increases in the sympathetic nervous system activity relative to work matched free flow exercise, this may precipitate adverse cardiovascular or cerebrovascular events, as BFR adds ∼5–10 mmHg to the usual blood pressure response during resistance training (Spranger et al., 2015; Cristina-Oliveira et al., 2020).

Other parts of the vascular system are similarly stressed during BFRT. A previous study demonstrated that BFRT in healthy subjects could acutely increase venous hypertension by ∼60 mmHg (Franz et al., 2020). While a healthy venous system can likely tolerate these increases with functioning venous valves in a longitudinal training program, patients with venous insufficiency or postoperative lymphedema might experience worsening of the cardiovascular health status induced by BFRT (Franz et al., 2020).

Results in the literature are mixed regarding exaggerated pressor responses, arterial blood pressure responses and autonomic modulation. With respect to the exercise pressor reflex, some studies evaluated the acute and chronic effects of BFRT and heart-rate variability and hemodynamic responses. The acute responses displayed in healthy older adults demonstrated that high intensity aerobic exercise (70% of VO_2_max) increased sympathetic-vagal balance and delayed vagal modulation at post-30 min recovery when compared to low load BFRT (using 50% AOP) (Ferreira et al., 2017). Also, a study using BFRT following an acute bout of bench-press exercise displayed decreased vagal modulation post-30 min for both low load BFRT (using rating of tightness at “7 of 10”) and high load but with a significant reduction after high load compared to low load (Tai et al., 2019). Furthermore, a previous study demonstrated that BFRT with 60% AOP compared to 80% AOP resulted in a reduction of SBP and DBP and increased vagal modulation after 48 h of an acute exercise session in older women with metabolic syndrome (Maciel et al., 2020). Conversely, BFRT with 80% AOP increased SBP by 15 mmHg immediately after the exercise session, possibly associated with higher metabolic stress during exercise and greater stimulation of the exercise pressor reflex. More research is needed to link BFRT application parameters along with responses to various loads with magnitude of exercise pressor responses in healthy and at-risk populations.

Regarding autonomic modulation and recovery, a previous study showed that heart rate variability (HRV) was delayed and accompanied by a significant reduction over time after single unilateral high intensity leg press exercise session compared to BFRT (Okuno et al., 2014). These results suggest a greater blunted parasympathetic recovery compared to BFRT (using an arbitrary pressure of 100 mmHg). The elevated lactate levels observed during high intensity exercise displayed a negative correlation with HRV, suggesting a blunted parasympathetic recovery [e.g., the square root of the mean of the sum of the squares of differences between adjacent NN intervals (RMSSD) and high frequency (HF) indices] compared to BFRT. The chronic effects of BFRT compared to traditional high load resistance training on HRV in inactive older adults (some of them with DM, hypertension, dyslipidemia, and venous insufficiency) demonstrated that after 12 weeks of resistance training, no training-related changes between groups were observed in HRV (Lopes et al., 2021). Nevertheless, only BFRT induced decrements of approximately seven mmHg for SBP and five mmHg for DBP. Hence, blood pressure reduction in the BFRT group may have resulted from vascular mechanisms such as angiogenesis and improved endothelial function despite potential acute increases in hemodynamic responses during exercise.

The effect of BFRT on angiogenesis or relevant transcription factors has been shown in both acute and longitudinal studies. Following an acute bout of exercise, BFRT increased vascular endothelial growth factor (VEGF), hypoxia-inducible factor 1 alpha (HIF-1α), isoforms nitric oxide synthase (eNOS), and VEGF receptors as kinase insert domain receptor (KDR) (Larkin et al., 2012; Ferguson et al., 2018). In addition, the acute effects of BFRT increase angiotensin-converting enzyme 2 (ACE2) and bone marrow-derived CD34 + hematopoietic stem/progenitor cells, markers associated with positive vascular and muscle health (Joshi et al., 2020). Furthermore, angiogenic adaptations following 3 weeks of BFRT have been shown to increase capillary number per myofiber and capillary area (Nielsen et al., 2020). Another study displayed increased reactive hyperemia index and transcutaneous oxygen pressure in the foot after 4 weeks of BFRT, indicating improved peripheral blood circulation compared to non-BFRT in healthy older adults (Shimizu et al., 2016).

However, positive effects on angiogenesis may ultimately depend on balancing training volume and frequency. A previous study demonstrated an adverse effect of low load resistance training with BFR (using a protocol of multiple sessions of BFRT per day) (Nielsen et al., 2020). A few participants displayed a transient thickening of the perivascular basal membrane while five participants showed basal lamina thickening 10 days after cessation of the intervention. Thickening of the perivascular basal membrane is associated with diseases like hypertension, peripheral artery disease, DM, inflammation, and miscellaneous disorders (Baum and Bigler, 2016). Triggers include high hemodynamic forces, congestion of venous blood flow and chronic hypoxia (Baum and Bigler, 2016). Degeneration of pericytes accompanying thickening of the basal lamina increases the distance of oxygen and nutritional substrates required to reach muscle fibers and may impair contractile activity during exercise caused by the limited energy supply (Baum and Bigler, 2016). Another study demonstrated that 4 non-failure sets of low load knee extension exercise with BFR (at 60% AOP) mitigated the increase of circulating endothelial progenitor cells (CD34 + VEGFR2 + and CD34 + CD45dimVEGFR2 +) in healthy male adults (Montgomery et al., 2019). In contrast, exercise without BFR resulted in a statistically significant increase in circulating endothelial progenitor cells (Montgomery et al., 2019). Although speculative, there may be an influence on the cellular responses observed that is dependent on individual characteristics, the BFRT protocol used and/or the timing of the blood draws.

Considering the information presented, in the presence of any of the following risk factors cited in Table 5, the patient with hypertension is classified as high risk (Fletcher et al., 2001; Milech et al., 2016; Diabetes, 2019; Mach et al., 2020), precluding the use of BFRT without physician clearance.

**TABLE 5:** Relevant risk factors concerning cardiovascular and hypertensive clients/patients before BFRT.

Patient’s Name:______________________________________________________________________________ Age:________________________________________________________________________________________ Sex:________________________________________________________________________________________
□ Acute myocarditis; □ Acute systemic illness; □ Angina or ischemic ST depression at a workload < 6 METs; □ Angiotomography of the coronary arteries with the presence of plaque; □ Aortic syndrome or venous thromboembolism; □ Cardiomyopathy with ejection fraction < 30%; □ Class III or IV heart failure; □ Complex ventricular arrhythmias not well controlled; □ Congenital heart disease; □ Coronary revascularization (percutaneous coronary interventions, coronary by-pass graft surgery, and other arterial revascularization procedure); □ Electrocardiographic alterations at rest or during effort; □ Endocarditis, or pericarditis; □ Exercise capacity < 6 METs; □ Fall in systolic blood pressure below resting levels during exercise; □ Familial hypercholesterolemia with atherosclerotic cardiovascular disease or with another major risk; □ Marked elevated single risk factors, in particular total cholesterol (>310 mg/dL), LDL-C (>190 mg/dL), or blood pressure ≥ 180/110 mmHg; □ Multivessel coronary disease with two major epicardial arteries having 50% of stenosis; □ Myocardial infarction and unstable angina; □ Non-sustained ventricular tachycardia with exercise; □ Peripheral artery disease; □ Postural hypotension (20 mmHg drop in systolic blood pressure with symptoms of dizziness or light-headedness); □ Presence of abdominal aortic aneurysm; □ Previous episode of primary cardiac arrest (e.g., cardiac arrest that did not occur in the presence of an acute myocardial infarction or during a cardiac procedure); □ Recent myocardial infarction < 3 months; □ SBP ≥ 160 mmHg and/or DBP ≥ 100 mmHg prior to exercise; □ SBP between 130 and 139 mmHg or DBP between 85 and 89 mmHg with target organ damage^b^, chronic kidney disease or diabetes mellitus; □ SBP between 140 and 159 mmHg or DBP between 90 and 99 mmHg with the presence of three or more cardiovascular risk factors^a^, with target organ damage^b^, chronic kidney disease or diabetes mellitus; □ SBP between 160 and 179 mmHg or DBP between 100 and 109 mmHg with the presence of 1 cardiovascular risk factor^a^, with target organ damage^b^, chronic kidney disease or diabetes mellitus; □ Self-reported easy bruising (Ballas and Kraut, 2008); □ Severe and/or symptomatic valve disease; □ Severe pulmonary hypertension; □ Stable angina; □ Stroke; □ Transient ischemic attack; □ Uncontrolled dysrhythmias; □ Unstable angina; □ Valvular heart disease with severe and asymptomatic valvular stenosis or regurgitation; □ Other medical condition that could be aggravated by exercise; □ A medical problem that the physician and BFR-user believe may be life-threatening;

*^a^Men ≥ 55 years or women ≥ 65 years; History of premature CVD in 1st degree relatives: men < 55 years old or women < 65 years old; Smoking; Dyslipidemia: total cholesterol > 190 mg/dL and/or LDL-cholesterol > 115 mg/dL and/or HDL-cholesterol < 40 mg/dL in men or < 46 mg/dL in women and/or Triglycerides > 150 mg/dL; Insulin resistance: fasting plasma glucose between 100 and 125 mg/dL, oral glucose tolerance test between 140 and 199 mg/dL in 2 h, glycated hemoglobin between 5.7 and 6.4%; Obesity: BMI ≥ 30 kg/m^2^, waist circumference ≥ 102 cm for men or ≥ 88 cm for women. ^b^Left ventricular hypertrophy; Carotid artery intima-media thickness > 0.9 mm or carotid plaque; Carotid-femoral pulse wave velocity > 10 m/s; Ankle-brachial index < 0.9; Stage 3 chronic kidney disease; Albuminuria between 30 and 300 mg/24 h or albumin-creatinine ratio urinary 30–300 mg/g. SBP, systolic blood pressure; DBP, diastolic blood pressure; CVD, cardiovascular disease; LDL, low density lipoprotein; HDL, high density lipoprotein; BMI, body mass index. Adapted from previous studies (Fletcher et al., 2001; Milech et al., 2016; Diabetes, 2019; Mach et al., 2020; Pelliccia et al., 2021).*

## Cardiovascular Disease and Blood Flow Restriction Training

Few studies have reported the effect of BFRT on patients with cardiovascular disease. Thus far, only 4 pilot studies (1 acute, 3 longitudinal) have been performed. The first study evaluated the post-exercise hemostatic and inflammatory responses of a BFRT session in 9 patients with stable ischemic heart disease (Madarame et al., 2013). The protocol consisted of four sets of knee extension exercise at an intensity of 20% 1RM (30-15-15-15 repetitions) using an arbitrary applied pressure of 200 mmHg. Sympathetic responses were heightened in the BFRT condition as evidenced by a significant increase in mean difference of noradrenaline (1.04 nmol/L^–1^) over the non-BFRT group. Irrespective of group allocation, post-exercise mean levels of D-dimer (0.07 μg/mL^–1^) and serum CRP increased compared to pre-exercise (51.3 ng/mL^–1^) baseline values, indicating a lack of an effect of BFRT on these hemostatic and inflammatory markers. However, D-dimer levels remained within a clinically normal range (<500 ng/mL) (Halaby et al., 2015), supporting the acute safety profile of BFRT in this small sample of patients.

The second pilot study evaluated the effects of 3 months of BFRT in 21 patients who underwent cardiovascular surgery (Ogawa et al., 2021). Patients were randomly assigned to a standard cardiac rehabilitation program (30 min of aerobic exercise twice a week for 3 months within the anaerobic threshold on a cycle ergometer) and resistance training with BFR (*n* = 11) or without BFR (*n* = 10) on leg extension and leg press starting at post-op day 5–7. Cuff pressure in the BFRT group was set at 100 mmHg and gradually increased to 160–200 mmHg over 2–3 weeks. During training, vital signs, electrocardiogram and rate of perceived exertion were continuously monitored. All patients received warfarin to reduce risk of post-surgical acquisition of a DVT with researchers closely controlling the prothrombin time-international normalized ratio. Markers of muscle damage (creatine phosphokinase), DVT markers (D-dimer) and adverse events were monitored in all patients. After 3 months, levels of muscle damage and D-dimer were within standard ranges and no adverse events were reported in the BFRT group, supporting its safety in the short-to-medium term post-surgery in cardiac rehabilitation patients. Paired with greater improvements in muscle mass [+20% (BFRT) vs. −4.3% (no BFRT)], muscle strength [+37% (BFRT) vs. + 9.1% (no BFRT)] and physical function [+26.4% (BFRT) vs. −5.4% (no BFRT)], this study supports the early integration of BFRT in this patient cohort (Ogawa et al., 2021).

The third pilot study evaluated the effects of 8 weeks of BFRT on leg extension strength (30% of 1RM with cuff inflated between 15 and 20 mmHg greater than brachial systolic pressure) along with hemodynamic, vascular function, and blood markers in patients with stable coronary artery disease (>3 months after acute coronary syndrome, revascularization, and/or documented by coronary angiography) (Kambic et al., 2019). Similar to literature on healthy individuals and other clinical populations (Hughes et al., 2017), BFRT increased muscle strength [+16.37% (BFRT) vs. + 5.29% (control group)] with reductions in SBP (−7 mmHg) and a tendency of better endothelial function (*p* = 0.079) (evaluated by FMD).

In addition, the same research group using the previous protocol evaluated the effect of 8 weeks of BFRT on hemodynamic and hemostatic markers in patients with stable coronary artery disease (>3 months after acute coronary syndrome, revascularization, and/or documented by coronary angiography). BFRT decreased SBP (−7 mmHg) compared to control group with no alterations on N-terminal prohormone B-type natriuretic hormone (212 ng/L), fibrinogen (2.94 g/L), and D-dimer levels (308 μg/L). Displaying no hazardous augmented hemodynamic response and beneficial effects on coagulation biomarkers, this study supports that use of BFRT can reduce cardiac stress through reductions in SBP without negatively altering clotting pathways (Kambic et al., 2021).

However, considering the aforementioned interventions were pilot studies, applying results to all ischemic and coronary artery diseases patients warrant caution. Particular concern should be given to acute sympathetic responses (noradrenaline) and the influence of a likely altered exercise pressor response in a potentially compromised cardiac system (Takano et al., 2005; Madarame et al., 2008; Shimizu et al., 2016). Thus, in the presence of any of the following risk factors cited above, the patient with concomitant hypertension and cardiovascular disease should likely avoid BFRT and another modality should be incorporated into the plan of care (Madarame et al., 2013; Malachias et al., 2016; Pinto et al., 2018; Kambic et al., 2019, 2021). We recognize this may be perceived as overly cautious, but clinicians are encouraged to actively collaborate with members of the medical team to ultimately determine BFRT candidacy if the patient may benefit from BFRT yet appears to have one or more risk factors present.

Risk stratification for DVT (Tables 2, 3) in conjunction with the table below are strongly encouraged to be included in the decision-making risk assessment (Table 5). The clinician should use sound judgment, have knowledge of relevant risk factors, and draw on experience to reduce risk to the patient when integrating BFRT. Strategies to theoretically reduce risk to the patient are similar to those just beginning BFRT and have been discussed elsewhere (Rolnick et al., 2021).

## Rheumatoid Arthritis and Blood Flow Restriction Training

BFRT has also been applied in autoimmune diseases like rheumatoid arthritis (RA). The first randomized controlled clinical trial evaluated the effects of BFRT (at 70% of AOP) after 12 weeks (twice a week) compared to high load RT in muscle strength, muscle mass, physical function, and quality of life. Similar increases in muscle strength [24.2% (high load RT) vs. 23.8% (BFRT)], muscle mass [10.5% (high load RT) vs. 9.5% (BFRT)] and physical function tests [14.7% (high load RT) vs. 11.2% (BFRT)] were observed. Only BFRT showed significant improvements in short form 36 health survey (SF-36) domains as physical and bodily pain and a significant reduction in visual analog scale (VAS) were reported (Rodrigues et al., 2020). For side effects, one patient withdrew from the study due to exercise-induced patellofemoral pain in the high load RT. Additionally, eight patients reported knee pain in the high load RT, requiring reductions of the load and repetitions (Rodrigues et al., 2020).

The second randomized controlled trial evaluated the effects of 4 weeks (three times a week, using 50% AOP) of low load BFRT compared to low load exercise training without BFRT on side effects, perceived pain, general satisfaction, and muscle strength. The interventions produced similar side effects in both groups. However, a case of headache and a cramping tendency in the calf muscles in one participant in the BFRT group was observed. For VAS, no changes for both groups from baseline and between groups were observed. Also, participants showed good compliance with training. For strength measurements, both groups improved muscle strength [23.2% (BFRT) vs. 17.8% (no BFRT)] (Jonsson et al., 2021).

Nevertheless, the literature reports that autoimmune disease-associated hypertension, premature atherosclerosis, myocardial dysfunction, electrical abnormalities, valvular involvement, pericarditis, and congestive heart failure lead to increased cardiovascular disease in RA patients (Faccini et al., 2016; Buleu et al., 2019; Wolf and Ryan, 2019). The prevalence of hypertension in RA patients appears to be slightly higher (Panoulas et al., 2008). Medications such as non-selective non-steroidal anti-inflammatory drugs (NSAIDs), cyclo-oxygenase II inhibitors (coxibs), glucocorticoids (GC), and some modifying antirheumatic drugs (DMARDs) should be screened for before beginning BFRT as they may cause hypertension and interfere with its effective control (Panoulas et al., 2008).

Risk stratification for RA patients should include screening for DVT risk (Tables 2, 3) as these patients are at an elevated risk of VTEs, pulmonary embolisms and DVT formation compared to the general population (Li et al., 2021). In conjunction with DVT screening, in the presence of any of the following risk factors cited below without physician clearance, the patient with RA should avoid BFRT, and another modality should be used (Table 6).

**TABLE 6:** Risk factors concerning rheumatoid arthritis patients before BFRT.

Patient’s Name:______________________________________________________________________________ Age:________________________________________________________________________________________ Sex:________________________________________________________________________________________
□ Unstable angina; □ Electrocardiographic alterations at rest or during effort; □ Recent myocardial infarction < 3 months; □ Class III or IV heart failure; □ Uncontrolled dysrhythmias; □ Severe pulmonary hypertension; □ Severe and/or symptomatic valve disease; □ Aortic valve stenosis; □ Aortic aneurism; □ Raynaud’s phenomenon; □ Acute myocarditis; □ Endocarditis, or pericarditis; □ Aortic syndrome or venous thromboembolism; □ Acute systemic illness; □ Coronary calcium score > 10 Agatstone; □ Carotid plaque (intima-media thickness > 1.5 mm); □ Angiotomography of the coronary arteries with the presence of plaque; □ Presence of abdominal aortic aneurysm; □ Exercise capacity < 6 METs; □ Angina or ischemic ST depression at a workload < 6 METs; □ Fall in systolic blood pressure below resting levels during exercise; □ Non-sustained ventricular tachycardia with exercise; □ Previous episode of primary cardiac arrest (e.g., cardiac arrest that did not occur in the presence of an acute myocardial infarction or during a cardiac procedure); □ SBP ≥ 160 mmHg and/or DBP ≥ 100 mmHg prior to exercise; □ Uncontrolled hypertension (>180/110 mmHg); □ SBP between 160 and 179 mmHg or DBP between 100 and 109 mmHg with the presence of 1 cardiovascular risk factor^a^, with target organ damage^b^, chronic kidney disease or diabetes mellitus; □ SBP between 140 and 159 mmHg or DBP between 90 and 99 mmHg with the presence of three or more cardiovascular risk factors^a^, with target organ damage^b^, chronic kidney disease or diabetes mellitus; □ SBP between 130 and 139 mmHg or DBP between 85 and 89 mmHg with target organ damage^b^, chronic kidney disease or diabetes mellitus; □ Postural hypotension (20 mmHg drop in systolic blood pressure with symptoms of dizziness or light-headedness); □ Marfan’s syndrome; □ Prednisolone > 5 mg/day over the past 3 months; □ Self-reported easy bruising (Ballas and Kraut, 2008); □ Other medical condition that could be aggravated by exercise; □ A medical problem that the physician and BFR-user believes may be life-threatening;

*^a^Men ≥ 55 years or women ≥ 65 years; History of premature CVD in 1st degree relatives: men < 55 years old or women < 65 years old; Smoking; Dyslipidemia: total cholesterol > 190 mg/dL and/or LDL-cholesterol > 115 mg/dL and/or HDL-cholesterol < 40 mg/dL in men or < 46 mg/dL in women and/or Triglycerides > 150 mg/dL; Insulin resistance: fasting plasma glucose between 100 and 125 mg/dL, oral glucose tolerance test between 140 and 199 mg/dL in 2 h, glycated hemoglobin between 5.7 and 6.4%; Obesity: BMI ≥ 30 kg/m^2^, waist circumference ≥ 102 cm for men or ≥ 88 cm for women. ^b^Left ventricular hypertrophy; Carotid artery intima-media thickness > 0.9 mm or carotid plaque; Carotid-femoral pulse wave velocity > 10 m/s; Ankle-brachial index < 0.9; Stage 3 chronic kidney disease; Albuminuria between 30 and 300 mg/24 h or albumin-creatinine ratio urinary 30–300 mg/g. SBP, systolic blood pressure; DBP, diastolic blood pressure; CVD, cardiovascular disease; LDL, low density lipoprotein; HDL, high density lipoprotein; BMI, body mass index. Adapted from previous studies (Fletcher et al., 2001; Milech et al., 2016; Diabetes, 2019; Mach et al., 2020; Rodrigues et al., 2020; Jonsson et al., 2021; Pelliccia et al., 2021).*

## Chronic Kidney Disease and Blood Flow Restriction Training

BFRT may become a potentially valuable tool for chronic kidney disease (CKD) patients as they present low tolerance to heightened perceptual demands of exercise training (Correa et al., 2021a,b; de Deus et al., 2021; Deus et al., 2022). Randomized controlled trials using the same protocol compared the effects of 6 months of periodized resistance training with and without BFRT 3 days a week in male and female patients with stage 2 CKD with hypertension and DM. The training included eight exercises: bench press, seated row, shoulder press, triceps pulley, barbell curls, leg press 45°, leg extension, and leg curl. Fifty percent (50%) of AOP was applied for the upper and lower limbs in a continuous application method during all exercise training sessions. Chronic effects of resistance training in this population from BFRT compared to traditional training include increased angiotensin 1-7, NO2-, increased antioxidant defense, decreased pro-oxidative markers, increased catalase activity, improved glucose homeostasis and hormones mediators of glucose uptake, and improved cardiac autonomic function (Correa et al., 2021a,b; de Deus et al., 2021; Deus et al., 2022). At the same time, BFRT diminished vasopressin levels and attenuated the decrease of estimated glomerular filtration, implying positive post-exercise benefits. Also, improved uremic parameters and inflammation profile (e.g., interleukin-6, IL-10, IL-15, IL-17a, IL-18, klotho, C-reactive protein, and tumor necrosis factor-alpha) was observed in the BFRT group indicating downregulation of inflammation-related markers and a decreasing of fibroblast growth factor 23 (marker of renal deterioration)-klotho axis (Correa et al., 2021a,b; Deus et al., 2022).

Other studies using BFRT on CKD patients demonstrated health benefits in end stage CKD (Cardoso et al., 2020). One 12-week randomized clinical trial evaluated the effect of intradialytic exercise with BFR (cycle ergometry during hemodialysis sessions three times a week) compared to conventional exercise on walking endurance test. Fifty percent of AOP was applied to the lower limbs continuously during all training sessions. Results of this study demonstrated no differences in functional capacity (walking distance in 6 min) between groups despite a larger improvement in walked distance in the BFRT group. BFR (at 50% of AOP) was applied during cycling exercise for 20 min within the first 2 h of hemodialysis with rate of perceived exertion of 12–13 (RPE Borg scale). Also, BFRT was shown effective as exercise alone in improving hemodialysis adequacy (e.g., how well blood is being cleansed), displaying a positive result in single-pool Kt/V-urea, equilibrated Kt/V-urea, urea reduction ratio, and urea rebound (Dias et al., 2020).

For adverse reports using BFR in CKD patients, a randomized clinical trial examined patients in three conditions using a cycle ergometer with bilateral BFR—non-BFR exercise during dialysis, BFR exercise off dialysis, and BFR exercise during dialysis (5 min of warm-up followed by two bouts of 10 min of cycling separated by 20 min of rest at 50% AOP). One case of exercise-related syncope (systolic and diastolic blood pressure of 88 and 68 mmHg, respectively) occurred with BFRT during hemodialysis. However, the participant chose to remain enrolled in the study. Also, in the same study, one additional instance of a participant feeling “light-headed” in recovery was reported. However, this was self-resolving, and ultrafiltration resumed within 5 minutes. Another randomized clinical study compared the effects of 8 weeks of exercise training including tennis ball, dumbbell weights, and handgrip exercise five times a week between BFRT group and exercise training alone observed isolated reports of tingling and fatigue in the upper limb of some patients who underwent BFRT at the time of exercise; however, these complaints were not sufficient for them to withdraw from the study (Silva et al., 2021).

Thus, BFRT may be a promising strategy for CKD patients to improve physical functioning and medical management of their condition. However, CKD patients are considered in the highest group for subsequent cardiovascular disease events (Levey et al., 1998). Traditional risk factors as advanced age, hypertension, hyperlipidemia, diabetes, and physical inactivity in conjunction with the patient characteristics of CKD (e.g., proteinuria, increased extracellular fluid (ECF) volume, electrolyte imbalance, anemia, and elevated thrombogenic factors) can help explain the excessive risk for CVD, and all appear in a greater proportion of CKD patients than the general populace (Levey et al., 1998). Besides, BFRT raises concerns as most stage III and IV CKD patients demonstrate hypertension and enhanced sensitivity of vascular α1-adrenergic receptors that may explain the more significant blood pressure reactivity with exercise (Sprick et al., 2019).

Considering that α1-adrenergic receptors are the primary mediators of vasoconstriction in response to catecholamines, BFRT has been shown to significantly increase plasma concentrations of adrenaline over similar free flow exercise (Takano et al., 2005; Madarame et al., 2008; Shimizu et al., 2016; Sprick et al., 2019). Patients with CKD already present a more remarkable rise in norepinephrine levels during exercise than controls and an exaggerated rise in SBP during moderate static handgrip compared to controls (Kettner et al., 1984; Park et al., 2008). Therefore, the acute increase in noradrenaline for CKD patients with additional cardiovascular comorbidities needs careful attention as an exaggerated exercise pressor response could predispose this population to increased risk of negative cardiovascular events (Spranger et al., 2015; Cristina-Oliveira et al., 2020).

Although BFRT in chronic kidney disease (CKD) is a promising strategy to improve physical functioning and medical management of the condition, there exist some potential concerns regarding the widespread application of BFRT in Stage III/IV CKD patients. However, BFRT in end-stage renal disease, stage two CKD patients, hypertensive patients in stage II CKD, and patients on hemodialysis (Cardoso et al., 2020; Dias et al., 2020; Correa et al., 2021a,b) appear to display no adverse effects. Of note, all included CKD studies present stringent exclusion criteria (e.g., cardiovascular events in the last 3 months, acute infection, neoplastic process, pregnancy, inadequate blood pressure control displayed by SBP above 180 mmHg and/or DBP above 105 mmHg, heart rate above 120 bpm during hemodialysis, decompensated patients, diabetes mellitus, symptomatic heart failure; history of nephrolithiasis or coagulation, human immunodeficiency virus infection, surgery within the past 3 months, drug or alcohol abuse, pre-exercise BP above 160/100 mmHg, previous diagnosis of coronary artery disease, and admission to an intensive care unit).

Considering this, we encourage using Tables 3–6 for safety purposes and previous critical contraindications to exercise for CKD patients as electrolyte abnormalities, recent changes in the electrocardiogram, excess of inter-dialytic weight gain > 4 kg since the last dialysis or exercise session, unstable on dialysis treatment, changing medication regime, pulmonary congestion, and peripheral edema (Smart et al., 2013). It is important to emphasize that clinicians must ensure that BFRT exercises be individualized to the patient with CKD’s current stage of physical ability. Patients in long-term dialysis are more prone to intense pain, musculoskeletal disorders, fragility fractures, and stable angina (Fry et al., 2008; Chan et al., 2009; Heaf et al., 2012). Thus, these patients should be gradually exposed to BFRT. With continued follow-up monitoring and application adjustments along the way (Rolnick et al., 2021) to reduce attrition secondary to increased perceptual and hemodynamic demands, patients with CKD can likely expect to improve their overall health and functionality with longitudinal BFRT programs. Collectively, the complexity and profound variability in CKD highlights the need to include multidisciplinary teams working to optimize individualized BFR management and determine the best progressions to maximize function and medical status.

## Anabolic Steroids, Ergogenic Substances, and Blood Flow Restriction Training

In bodybuilders and those pursuing physique-related sports, the literature demonstrates the potential applicability of BFRT. Two recent reviews have highlighted the theoretical benefits of BFRT in bodybuilders during resistance and aerobic training during contest preparation (Rolnick and Schoenfeld, 2020a,b). While the use of anabolic steroids (AS) appears to be higher in those pursuing physique-related endeavors (Haerinejad et al., 2016), AS use has been reported in high school athletes (Windsor and Dumitru, 1989) and men aiming to improve body image (Kanayama et al., 2020) indicating that AS use is not just for bodybuilding. However, AS use increases the likelihood of adverse effects such as dyslipidemia, polycythemia, hyperhomocysteinemia, a hypercoagulability state, cardiac and vascular hypertrophy, ventricular arrhythmias, impaired angiogenesis, redox balance, arterial hypertension, and cardiomyocyte apoptosis (Patane et al., 2020; Seara et al., 2020). Also, it appears that the long-term effects of AS may not be reversible (Seara et al., 2020), increasing risk during BFRT despite cessation and their apparently healthy appearance.

In addition, most AS users tend to use multiple substances at once, causing synergic effects and systemic disorders whose cause cannot be readily identified by the clinician (Pope et al., 2014). The use of AS combined with other compounds like thyroid hormones, growth hormone, insulin, diuretics, caffeine, yohimbine, and sympathomimetics products (e.g., ephedra alkaloid-containing products) (Cimolai and Cimolai, 2011; Mancano, 2015; Spranger et al., 2015; Seara et al., 2020) may enhance the concern of integrating BFRT into a strength training program by increasing risk of adverse events. Although BFRT appears to be a feasible strategy for maximizing hypertrophy gains in bodybuilders during the pre-contest period, these athletes enter a negative energy balance and dehydration linked to restrictive diets (Alves et al., 2020). The same strategy of negative energy balance occurs in other sport disciplines associated with pre-contest periods, such as in Taekwondo (Rhyu et al., 2014). Such strategies might initiate negative mood alterations, autonomic deregulation, compromised force-generating capacity, as well as elevations in cortisol and reductions in testosterone levels (de Moraes et al., 2019). Moreover, the pre-contest period might induce damage to cell components and greater severity of upper respiratory tract infections from an increase of inflammatory mediators and pro-oxidant markers (Fry et al., 2008).

Recently, a study showed that severe restriction energy intake during the pre-contest period was associated with an increase in oxidative stress markers (TBARS, malondialdehyde and protein carbonyls), impaired upregulation of antioxidant enzymes (glutathione reductase, catalase activity, and superoxide dismutase), and decreased plasma total antioxidant capacity (Rhyu et al., 2014; de Moraes et al., 2019). Moreover, a previous study revealed that during the pre-contest preparation period, different strategies (AS, clenbuterol, thyroid hormone, and ephedrine) might result in maladaptive effects on the lipid profile and alteration of transaminases, increasing the atherosclerotic heart disease risk and liver dysfunction (de Souza et al., 2018). Thus, these adverse effects must be considered in critical periods of bodybuilder preparation (pre-contest) because the underlying systemic changes may potentiate the possible adverse responses related with BFRT.

Furthermore, adverse events associated with ergogenic aids (e.g., dietary supplements) should be considered in the screening process (Geller et al., 2015). Weight loss supplements are implicated in up to 25% of emergency department visits (e.g., palpitations, chest pain, tachycardia, syncope, headache) followed by energy supplements (Geller et al., 2015). Besides, per year females visit the emergency department almost three times that of male patients. In addition, stimulants found in dietary supplements as 1,3-dimethylamylamine, ephedra, β-methylphenethylamine, N,α-diethyl-phenylethylamine, N-caffeoyldopamine, and N-coumaroyldopamine are associated with potential adverse reactions including arrythmias and myocardial infarction and should be considered in screening (Cohen, 2014).

Creatine is another frequently used weightlifting supplement that may heighten risk of adverse events as some published case reports displayed associations between creatine supplementation and venous thrombotic events (Mancano, 2014; Tan et al., 2014; Moussa and Chen, 2021). The possible explanations include osmotic changes secondary to increased intracellular creatine, drawing water into the muscle (Mancano, 2014). This could lead to dehydration, especially in hot environments and cases of heat stroke have been reported among users (Tan et al., 2014). Thus, use of creatine supplementation and the practice of dehydration and electrolyte manipulation in the final days prior to competition and use of BFRT may represent a concern given reported outcomes in the literature. However, no creatine-related adverse events have been reported in the literature either in healthy non-bodybuilders or bodybuilders.

Therefore, the information mentioned above only provides context to potentially relevant precautions when weighing the safety risk for BFRT. Differences due to administration dosage, pattern, and the use of several AS, and ergogenic substances, simultaneously must be accounted for before BFRT, and likely a pre-screening question could aid in this determination.

The decision to use BFRT is based on carefully weighing the evidence for adverse events and providing the safest course of action. A complete blood count and a comprehensive metabolic panel including red and white blood cells, lipid profile, platelets, D-dimer, and fibrinogen can be especially relevant for screening to review the overall health of individual (if within scope of practice) before inclusion of BFRT, especially if AS and/or ergogenic substances use is suspected.

## Post-Surgery and Blood Flow Restriction Training

When to begin BFRT in the post-surgical patient is one of the most important clinical questions needed to be answered given the rapid effects of disuse on skeletal muscle mass, strength, and cardiovascular capacity. Some studies have shown the safety of the BFRT at various time intervals as early as 2 days post-surgery (Iversen et al., 2016). A previous study demonstrated safety in rehabilitation 2–3 weeks after anterior cruciate ligament surgery using BFRT (Hughes et al., 2019). Another study applied BFRT 3 weeks post- knee arthroscopy surgery (Tennent et al., 2017). One author group has had such success with BFRT that they apply BFRT to all major post-surgical knee patients after 3 weeks (Noyes et al., 2021).

Even with these findings, the use of BFRT following surgery must be carefully analyzed. The risk of DVT is increased 100-fold in the first 6 weeks following surgery (Bond et al., 2019) and pulmonary embolism risk is more significant in the 12 weeks following surgery in middle-aged women, which of course, will depend on the type of surgery (Sweetland et al., 2009). The relative risk for thrombosis after hip and knee arthroplasty is 220 times higher in the first 6 weeks after surgery, 91.6 times higher after cancer surgery, and 87 times higher after vascular surgery highlighting that surgery of any kind increases risk of DVT formation (Sweetland et al., 2009).

Therefore, we recommend caution when using BFRT post-surgery with and without the use of thromboprophylaxis. Risk will also be modified based on the health status of the patient and any relevant comorbidities (e.g., diabetes mellitus, hypertension, or cardiovascular disease). Clinicians are recommended to screen for DVT from the modified Caprini thrombosis risk factor assessment or IMPROVE scale (Motykie et al., 2000; Caprini, 2005; Rosenberg et al., 2014; Golemi et al., 2019) with any post-surgical patient. The reader is referred to Bond et al. (2019) for a more in-depth look at the post-surgical risk of acquiring a DVT with BFRT.

## Patients With COVID-19 and Blood Flow Restriction Training

Patients with COVID-19 that underwent mechanical ventilation likely have intensive care unit-acquired weakness and present with muscle degradation/atrophy (Barbalho et al., 2019; Lad et al., 2020). The changes seen in patients with critical illness myopathy that lead to intensive care unit-acquired weakness include severe muscle atrophy that affects both type I and type II fibers, along with preferential and significant loss of thick filament myosin protein, sarcomere disorganization and electrical hypoexcitability (Lad et al., 2020). Previous studies raised the possibility of using BFRT to counteract severe muscle atrophy and low muscle strength post-intensive care unit, possibly being able to provide a therapeutic alternative approach to traditional rehabilitation (Lad et al., 2020; Roman-Belmonte et al., 2020). Another study displayed that BFRT was able to reduce the magnitude of the rate of muscle wasting in elderly coma patients admitted to the intensive care unit (Barbalho et al., 2019). The 11-day protocol consisted of passive mobilization of 3 sets of fifteen repetitions of knee flexion-extension (at 80% of AOP) at a 2-s flexion/2-s extension cadence and was performed daily throughout the patient’s hospitalization. Thus, BFRT might be an essential aid for patients with COVID-19 admitted to the intensive care unit to prevent excessive muscle wasting (Barbalho et al., 2019). However, patients with recent COVID-19 infection display several laboratory abnormalities and thromboembolic complications such as elevated D-dimer, platelets activation, elevated levels of vWF, hyperviscosity and fibrinogen in the blood during the early stages of infection compared to healthy controls (Gupta et al., 2020). Also, alveolar-capillary microthrombi are 9 times more prevalent in individuals with COVID-19 than in those with influenza (Ackermann et al., 2020).

These hemostatic and inflammatory changes reflect endothelial damage. Further, COVID-19 is associated with harmful effects on many other organ systems such as heart, renal, dermatological, neurologic, renal, hepatic, endocrine, and gastrointestinal (Gupta et al., 2020). These systemic changes indicate a pro-inflammatory state and likely elevated risk for clotting. Risk of DVT appears high in patients with COVID-19, and intensive care unit patients with severe COVID-19 infections have been shown to have higher incidences of DVT compared to patients admitted in the general wards (Sarkar et al., 2021). The clinician must also be aware that patients with COVID-19 display common comorbidities as hypertension, DM, and cardiovascular disease (Huang et al., 2021). Hence, previous risks assessment models as Tables 2–6 should be used with these patients.

The information as mentioned earlier suggests that clinicians should consider (if available) (Gupta et al., 2020):

□ Disease severity: (1) not admitted to hospital with resumption of normal activities; (2) not admitted to hospital, but unable to resume normal activities; (3) admitted to hospital but not requiring supplemental oxygen; (4) admitted to hospital but requiring supplemental oxygen; (5) admitted to hospital requiring high-flow nasal cannula (HFNC), non-invasive mechanical ventilation (NIV), or both; (6) admitted to hospital requiring extracorporeal membrane oxygenation, invasive mechanical ventilation (IMV) (Huang et al., 2021).
□ Inflammatory markers: elevations in erythrocyte sedimentation rate, C-reactive protein, ferritin, IL-6, lactate dehydrogenase.
□ Coagulation indices: elevated D-dimer and fibrinogen; prolonged prothrombin time and partial thromboplastin time.
□ Consider if the patient used thromboprophylaxis post-hospitalization, particularly for those with a history of critical illness.

As these are not frequently known by the patient presenting to outpatient rehabilitation, collaborating with a referring physician is advised to determine if these values exceed normal. Finally, the clinician should have working knowledge and understanding of COVID-19 to mitigate risk using BFRT in this population. More studies are needed to make stronger BFRT recommendations with a sufficient degree of confidence.

## Apparently Healthy Individuals and Blood Flow Restriction Training

Most studies involving BFRT are published using apparently healthy individuals (Moriggi et al., 2015). Thus, considering the absence of adverse responses, the risk stratification includes the following items (Table 7).

**TABLE 7:** Risk factors concerning apparently healthy individuals before BFRT.

Patient’s Name:______________________________________________________________________________ Age:________________________________________________________________________________________ Sex:________________________________________________________________________________________
In the presence of any items in the list below, BFR training should be avoided. □ Self-reported easy bruising (Ballas and Kraut, 2008); □ Acute systemic illness; □ A medical problem that the physician and BFR practitioner believes may be life-threatening; **Note:** For apparently healthy individuals with < 2 (low cardiovascular risk) factors*^a^*. BFR training may be considered safe.

*^a^Men ≥ 55 years or women ≥ 65 years; History of premature CVD in 1st degree relatives: men < 55 years old or women < 65 years old; Smoking; Dyslipidemia: total cholesterol > 190 mg/dL and/or LDL-cholesterol > 115 mg/dL and/or HDL-cholesterol < 40 mg/dL in men or < 46 mg/dL in women and/or Triglycerides > 150 mg/dL; Insulin resistance: fasting plasma glucose between 100 and 125 mg/dL, oral glucose tolerance test between 140 and 199 mg/dL in 2 h, glycated hemoglobin between 5.7 and 6.4%; Obesity: BMI ≥ 30 kg/m^2^, waist circumference ≥ 102 cm for men or ≥ 88 cm for women. Adapted from previous studies (Fletcher et al., 2001; Milech et al., 2016; Diabetes, 2019; Mach et al., 2020; Pelliccia et al., 2021).*

Use of Caprini’s thrombosis risk factor assessment and modified IMPROVE risk score (Motykie et al., 2000; Caprini, 2005; Spyropoulos et al., 2011; Mahan et al., 2014; Rosenberg et al., 2014; Golemi et al., 2019; Tables 2, 3) should also be integrated into the screening process in conjunction with risk factors cited in Table 7. When in doubt, the clinician is advised to consult with other clinician experts/physicians to determine appropriate BFRT candidacy (Rolnick et al., 2021).

## Discussion

The danger associated with not exercising is more significant than that associated with exercising (Pedersen and Saltin, 2006), but the results of the studies displayed till now highlight that BFRT should be carefully implemented. The authors of this manuscript are aware that not all individuals can perform heavy load strength training and thus the clinician may decide to use with BFRT after evaluating risk factors. In that case, we recommend that coagulation indices as safety issues for DVT before and after regular BFRT and blood pressure monitoring be frequently analyzed if possible. We also recommend some criteria adapted from a previous study that can be used to stop training during BFRT (Fletcher et al., 2001; O’Brien et al., 2018):

• Development of significant ventricular or atrial arrhythmias.
• The onset of chest pain/discomfort, or other symptoms, suggestive of myocardial ischemia.
• Dizziness, confusion, deteriorating balance, or other significant neurological symptoms.
• Paleness or cyanosis.
• Vomiting, nausea, or feeling generally unwell.
• Decrease in systolic blood pressure from rest < 10 mmHg in the absence of symptoms.
• Hypertensive systolic blood pressure ≥ 250 mmHg and or diastolic blood pressure ≥ 115 mmHg.
• Exhaustion or fatigue (malaise), sometimes persisting for days, that is out of keeping with the person’s usual response to exercise at a given intensity.
• Swelling and shortness of breath.
• Skin of the affected limb that is too hot or cold to touch.
• Increased/excessive pain in the affected limb.
• Excessive discoloration of the affected limb.
• Subject requests to stop.

Clinicians that will use the proposed risk stratification in this manuscript should have education and training in pathological states and their significance to the BFRT response in both resistance and aerobic training approaches. Education and training can come from curated post-professional BFR courses or self-study. Both are likely very important to reduce risk of improper application of BFRT that may increase risk of adverse events and liability.

The *American Physical Therapy Association* defines scope of practice for physical therapists using a threefold definition: professional, jurisdictional, and personal (APTA, 2020). While jurisdictional scope of practice is regionally defined and professional scope is determined through accreditation and the licensing process to become a physical therapist, personal scope of practice is achieved through activities that the clinician is educated and trained on and is competent to perform. With respect to BFRT, competency likely includes knowledge of typical BFRT responses to exercise, characteristics of BFRT devices and ability to apply BFRT according to established guidelines. Thus, just because BFRT is within the scope of practice of physical therapists does not itself demonstrate competency to safely perform (CA.GOV, 2005).

The proposed risk stratification is not yet backed up by specific data regarding the clinical benefit and cost-effectiveness of BFRT. Hence, the risk stratification proposed herein reviews adverse effects displayed by data derived from expert opinions, small studies, randomized clinical trials, and non-randomized studies. Nevertheless, the proposed stratification risk score should be externally validated to demonstrate if it can be used with reproducible accuracy and confidence to ensure appropriate patient care before BFRT. However, we believe that this risk stratification assessment may represent a useful initial effort aimed at minimizing adverse responses in the clinical setting for clinicians looking to improve rehabilitation and fitness outcomes in their patients. The proposed risk stratification application into practice is not likely to be very long and cumbersome to complete (e.g., taking minutes to integrate). However, shorter risk stratifications have the obvious advantage of brevity and provide sufficient information on relative risk. Future risk stratification questionnaires can build upon the proposed risk factors in each condition to further reduce potentially redundant criteria such as in the IMPROVE scale (Rosenberg et al., 2014) to stratify the primary risk factors determined through longitudinal research. However, the lesson to be drawn from efforts to derive reliable and valid stratification risks is that substantial empirical work is needed to ensure that proposed risk stratifications operate as intended.

Finally, the authors attempted to summarize the strength of the available scientific evidence underlying BFRT observed in the various subsections of this narrative review. However, traditional narrative reviews and systematic reviews differ in several ways. Systematic reviews attempt to minimize bias by assessing the methodologic quality of included studies, inclusion and exclusion criteria, validity, and so forth (West et al., 2002). Significant challenges arise when evaluating the strength of evidence in a body of knowledge comprising combinations of observational and randomized clinical trial data (West et al., 2002). No single approach is ideally suited for assessing the strength of scientific evidence, particularly in cases where evidence is drawn from various methodologies (West et al., 2002). Considering that BFRT is a modality with growing interest in the last decade, especially for clinical patients, it would be too soon to summarize the strength of evidence for each subsection described within this manuscript. Furthermore, systematic reviews have a broad search and coding protocol to attend to a specific and narrow scope. Unlike narrative reviews, systematic reviews do not cover a broad topic that involves multiple independent variables with multiple and distinct outcomes. A narrative review does not use the systematic search and analytic protocols as a systematic review although it can cover a broader research topics hidden by multiple outcomes that follow BFRT intervention. This type of research is relevant to elucidate mechanisms and suggest potential gaps for further investigation in BFRT.

## Conclusion and Perspectives

Clinicians should use judgment, knowledge of relevant risk factors, clinical experience, and in some cases, common sense to provide adequate screening and monitoring when administering BFRT. In the final analysis, the inclusion to use BFRT is based on carefully weighing the evidence for adverse events and providing the safest course of action for the patient, allowing for optimization and better planning of care. Therefore, a risk stratification is likely essential for the start of a safe and effective BFRT program and can help in the decision-making process for all clinicians.

A useful BFRT risk stratification goes beyond the individual’s classification risk as it also allows the clinician to direct the therapeutic approach, establish a level of monitoring and the determine the appropriate dose of exercise. These proposed adapted risk stratifications can help in the screening process, removing barriers to initiating BFRT programs for clinicians in all settings. Finally, the risk stratification might serve as a guideline for clinical protocols and future randomized controlled trial studies.

## Author Contributions

DN and NR conceived, designed the study, and conducted the reference review. DN, NR, IN, RS, and FB contributed to the writing of the manuscript. All authors contributed to the article and approved the submitted version.

## Conflict of Interest

NR is the founder of THE BFR PROS, a BFR education company that provides BFR training workshops to fitness and rehabilitation professionals across the world using a variety of BFR devices. NR has no financial relationships with any cuff manufacturers/distributors. The remaining authors declare that the research was conducted in the absence of any commercial or financial relationships that could be construed as a potential conflict of interest.

## Publisher’s Note

All claims expressed in this article are solely those of the authors and do not necessarily represent those of their affiliated organizations, or those of the publisher, the editors and the reviewers. Any product that may be evaluated in this article, or claim that may be made by its manufacturer, is not guaranteed or endorsed by the publisher.
